# A Rare Case of Traumatic Bilateral Fibular Head Fractures

**DOI:** 10.1155/2010/920568

**Published:** 2010-08-08

**Authors:** Anastasios Chytas, Antonios Spyridakis, John Gigis, Theodoros Beslikas, Nikolaos Panos, John Christoforidis

**Affiliations:** 2nd Orthopaedic Department of Aristotle University of Thessaloniki, General Hospital of Thessaloniki “G. Gennimatas”, Ethnikis Aminis 41, Thessaloniki, PC 54635, Greece

## Abstract

Avulsion fibular head fractures are rare. There is only one reference of bilateral fibular fractures after epileptic seizure. We aim to present the mechanism and the treatment of this rare injury. We present the case of a 30-year-old woman who was hit by a car on the anteromedial side of both knees. Clinical and radiographic control showed bilateral fibular head fractures. Knee instability was not found at both knees and MRI did not show any concomitant ligament ruptures. Bone bruises of both medial condyles found in MRI explain the mechanism of this injury. The patient was treated conservatively with functional knee braces for 6 weeks allowing full range of motion, but otherwise mobilised as normal without any support. Six weeks after the trauma, there were no symptoms while the fractures sites had united completely after 6 months. One year postinjury the patient was free from symptoms.

## 1. Introduction

The fibula, due mainly to its anatomical position and function, is one of the most common sites of long bone fractures. Usually fibular fractures coexist with tibial fractures, being as a result of a high-energy pattern of injury [1]. Isolated fibular fractures are commonly located at the diaphysis and distal third. Fractures of the fibular head are uncommon and usually caused by a direct blow or a varus stress on the knee [2]. The most common mechanism is direct blow on the anteriomedial tibia with the knee in extension [3]. There is only one reference of bilateral fibular head fractures after epileptic seizure [4]. As far as we know this is the first report of traumatic bilateral fibular head fractures.

## 2. Case Presentation

**Figure 1:**
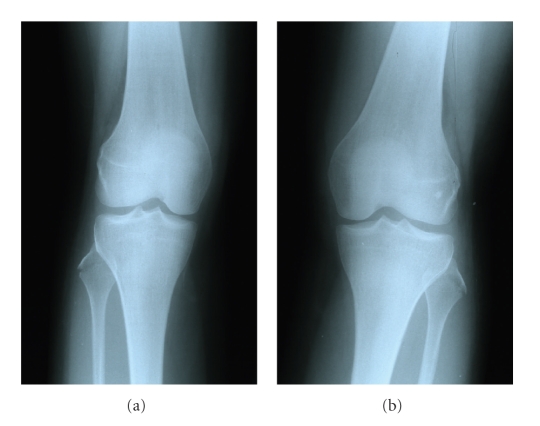
Anteroposterior radiographs of both knees immediately after trauma. Fractures of the lateral borders of both fibular heads are easily seen.

**Figure 2:**
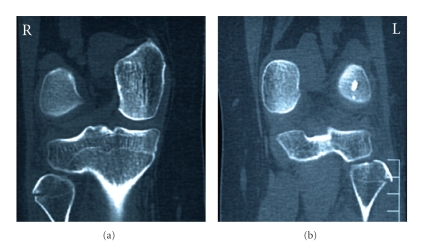
CT of both knees. The displacement of the fractures is minimal (2 mm for the right fibular head and 3 mm for the left).

**Figure 3:**
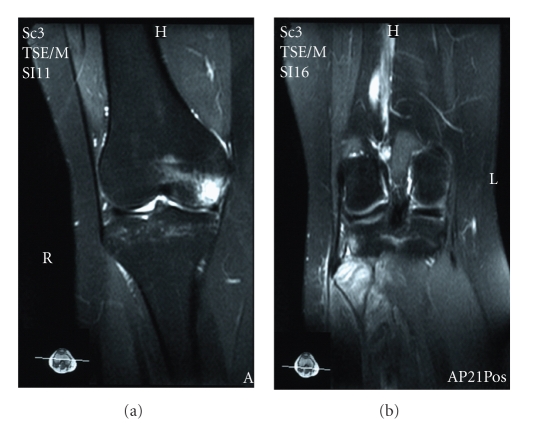
MRI of the right knee showing medial condyle bruise (a) as well as edema of the head of fibula due to fracture (b).

**Figure 4:**
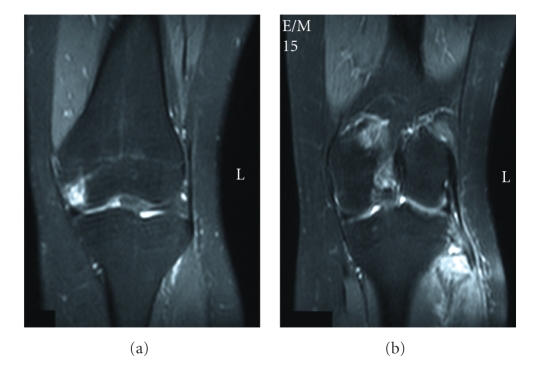
MRI of the left knee showing medial condyle bruise (a) as well as edema of the head of fibula due to fracture (b).

**Figure 5:**
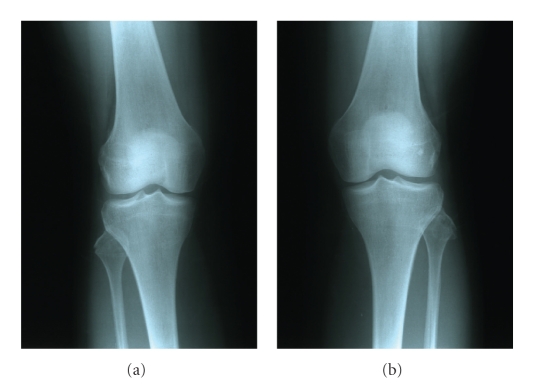
Anteroposterior radiographs of both knees 6 months after trauma. Fractures of both fibular heads are fully united.

We present a case of a 30-year-old woman who was hit by a car on the anteromedial side of both knees. She was transferred to our emergency department and her only symptom was bilateral lateral knee pain mostly during weight-bearing. Clinical examination showed bilateral lateral knee tenderness with erosions and mild effusions at the front of both knees. Knee instability was not found at both knees. There were no nerve dysfunctions or other concomitant injuries. The radiographic control showed bilateral fibular head fractures (Figure 1). We performed a CT scan of both knees to evaluate the displacement of the bone fragments (Figure 2). The lateral sides of fibular heads were avulsed, but the displacement was less than 3 mm at both sites. An MRI examination excluded ligamentous injuries and showed bone bruises at the medial femoral condyle of both knees (Figures 3 and 4). The patient was treated conservatively with functional knee braces for 6 weeks allowing full range of motion. Since medial femoral condyle bruises were mild and without any symptoms, full weight-bearing was permitted. Functional knee braces were used in order to eliminate the lateral knee patient during weight-bearing, although instability was not apparent. Six weeks after the trauma, there were no symptoms while the fractures sites had united completely after 6 months (Figure 5). One year postinjury the patient was free from symptoms.

## 3. Discussion

Avulsion fibular head fractures are rare. In a retrospective study of 2318 knee injuries, only 13 sustained this fracture (0.6%) [3]. Bilateral traumatic fibular head fractures have not been described before. The only reference is after epileptic seizure, where avulsion of both fibular heads occurred due to forceful contraction of the biceps femoris [4]. 

Several different types of avulsion fractures of the fibular head occur, however, related to the attachment sites of the many ligaments that attach to the fibular head. Anatomically, the lateral collateral ligament (FCL) and tendon of the long head of the biceps femoris muscle (BFT) are attached to the lateral margin of the fibular head. The popliteofibular, arcuate, and fabellofibular ligaments are attached to the fibular styloid process [5]. An avulsion fracture of the head of the fibula has been described as an important indicator of posterolateral instability of the knee. The “arcuate” sign refers to an avulsed bone fragment related to the insertion of the posterolateral corner ligamentous structures (BFT, FCL, arcuate ligament, popliteofibular ligament). A bony arcuate injury may be more readily appreciated on plain radiographs than on MRI, but the latter best assesses the extent of associated ligamentous injury [6]. In one series of 18 “arcuate” fractures seen on plain radiographs, all cases had avulsed fragments attached to the FCL, BFT, or both demonstrable on MRI [7]. In another study, the avulsion fracture of the styloid process of the fibular head was apparently related to injuries of the arcuate complex in all 13 patients [3]. Our patient had avulsion fractures at the lateral side of the fibular heads due to the avulsion of biceps femoris tendon and lateral collateral knee ligament. The attachments of popliteofibular, fabellofibular, and arcuate ligaments on the fibular styloid process were intact and thus, instability was not apparent at both knees. One biomechanical study considered the popliteofibular ligament to be the primary static stabilizer to the posterolateral corner of the knee [3]. 

A concomitant injury of either the anterior or posterior cruciate ligament in patients with an injury involving structures in the posterolateral aspect of the knee has been stressed in the literature [8–11]. When a patient sustains an injury to both the arcuate complex and a cruciate ligament, it may compromise the function and stability of the knee to a greater extent than an isolated injury to either structure alone [11]. MR images are important in such avulsion fractures of the fibular head because of the high incidence of concomitant knee ligament lesions. Our patient had no ligament ruptures but bilateral medial femoral condyle bone bruises. These bruises explain the mechanism of injury. She was hit at the anteromedial sides of her knees while her tibias were externally rotated and the knees, were extended. This is the common mechanism of fibula head fractures [3]. 

Although concomitant ligament ruptures and instability impose surgical treatment, isolated fibular head fractures may be treated with functional knee bracing for six weeks [12]. Due to bilateral fractures, we allowed our patient full weight-bearing with functional knee braces.
